# On the Value of the Hemorrhage in the Treatment of Wounds

**Published:** 1887-08

**Authors:** 


### On the Value of the Hemorrhage in the Treatment of Wounds.

Professor Turazza, in the *Gazetta degli Ospitali* of April 13, publishes a note showing that hemorrhage from wounds, when not due to the lesion of large vessels or carried to excess, is of small importance, and does not interfere with primary union. He believes that the rule generally laid down regarding the first dressing of a wound—viz., to secure complete arrest of hemorrhage and to apply firm compression—though excellent, is not important. Professor Turazza relies on perfect disinfection of the bleeding surface, as far as within reach, by means of weak solutions of phenic acid, or corrosive sublimate. He then leaves the cavity of the wound full of blood, without any doubt as to primary union, the edges of the wound being very accurately sutured. As the result of his experience he formulates a new rule in surgery—"that in wounds perfectly disinfected and free from foreign substances, the effusion of blood is not a source of danger, but the reverse; for the extravasated blood fills up the cavity of the wound perfectly, preventing the formation of empty spaces, and rendering both compression and drainage superfluous, and, further, the organization of the clot favors healing." Professor Turazza also expresses himself as decidedly averse to the use of the drainage-tube within the narrowest limits, and would deprecate its use even in ovariotomy, hysterectomy, and amputation of the breast, thinking it more dangerous than useful.

This is practically the method advocated by Schede, of Hamburg.

